# Hemodynamic and Metabolic Assessment of Neonates With Punctate White Matter Lesions Using Phase-Contrast MRI and T2-Relaxation-Under-Spin-Tagging (TRUST) MRI

**DOI:** 10.3389/fphys.2018.00233

**Published:** 2018-03-19

**Authors:** Ying Qi, Peiying Liu, Zixuan Lin, Hanzhang Lu, Xiaoming Wang

**Affiliations:** ^1^Department of Radiology, Shengjing Hospital of China Medical University, Shenyang, China; ^2^Department of Radiology, Johns Hopkins University School of Medicine, Baltimore, MD, United States

**Keywords:** punctate white matter lesions, PC MRI, TRUST MRI, CMRO_2_, CBF, Yv, OEF

## Abstract

The brain's hemodynamic and metabolism of punctate white matter lesions (PWML) is poorly understood due to a scarcity of non-invasive imaging techniques. The aim of this study was to apply new MRI techniques to quantify cerebral metabolic rate of oxygen (CMRO_2_), global cerebral blood flow (CBF), oxygen saturation fractions in venous blood (Yv) and oxygen extraction fraction (OEF) in neonates with PWML, for better understanding of the pathophysiology of PWML. Fifty-one newborns were recruited continuously, including 23 neonatal patients with PWML and 28 normal control neonates. Phase-contrast (PC) MRI and T2-Relaxation-Under-Spin-Tagging (TRUST) MRI were performed for the measurement of CBF and Yv. OEF and CMRO_2_ were calculated from the CBF and Yv values. The total maturation score (TMS) was assessed for each neonate on standard T1, 2-weighted images to evaluate cerebral maturation. The CMRO_2_, CBF, Yv, and OEF values were compared between groups, and their associations with age and TMS were evaluated. Significant differences between PWML group and control group were found in CMRO_2_ (*P* = 0.020), CBF (*P* = 0.027), Yv *(P* = 0.012), OEF (*P* = 0.018). After age/maturation is accounted for, Yv and OEF showed significant dependence on the groups (*P* < 0.05). Newborns with PWML had lower OEF and higher Yv. CMRO_2_, CBF and brain volume were correlated with age (*P* < 0.001) and TMS (*P* < 0.05). It is feasible to use non-invasive MRI methods to measure cerebral oxygen supply and consumption in neonates with PWML. Newborns with PWML have lower oxygen consumption. Yv and OEF may be helpful for the diagnosis of PWML. The positive correlation between CBF and TMS, and between CMRO_2_ and TMS suggested that as myelination progresses, the blood supply and oxygen metabolism in the brain increase to meet the escalating energy demand.

## Introduction

Punctate white matter lesions (PWML) are frequently recognized on magnetic resonance imaging (MRI) in unmyelinated white matter as hyperintensity on T1-weighted images with or without hypointensity on T2-weighted images (Niwa et al., 2011). The incidence of PWML in preterm newborns with low and extremely low birth weight is 50% (Volpe, 2003). Among survivors, 5–10% exhibit cerebral palsy and 50% have deficits in cognition, behaviors or attention (Wilson-Costello et al., 2007). Several studies have raised the hypothesis that PWML may be related to the milder forms of cognitive and behavioral problems found at school age (Miller et al., 2005; Sie et al., 2005), but little is known about the brain function in PWML neonates. Quantitative evaluation of brain hemodynamics and cerebral oxygen metabolism may provide important functional information for the understanding of neonatal PWML.

Cerebral blood flow (CBF) as a measure of brain perfusion, is an important indicator of brain function. Currently there are several techniques used to measure CBF, including computed tomography (CT) perfusion (Dani et al., 2012), positron emission computed tomography (PET) (Altman et al., 1993; Wright et al., 2016), vascular ultrasound (Burgess et al., 2018), ^133^xenon clearance (Colditz et al., 1988) and MRI (Wang and Licht, 2006; Dai et al., 2008). CT, PET, and ^133^xenon clearance need contrast agents or tracers, which usually are radioactive and therefore are not typically used in newborns. Vascular ultrasound can be used to measure blood flow in internal carotid arteries (ICA), but is difficult to assess vertebral arteries (VA), which are also feeding arteries to the brain, due to the blockage by bones. In MRI techniques, arterial spin labeling (ASL) (Massaro et al., 2013; Boudes et al., 2014; De Vis et al., 2014; Ouyang et al., 2017) and phase-contrast (PC) MRI (van Kooij et al., 2010; Benders et al., 2011; Varela et al., 2012; Jain et al., 2014; Liu et al., 2014) are two techniques that measure CBF without exogenous contrast agent and have been utilized in newborns. However, in neonates, ASL technique suffers from low signal-to-noise ratio and sensitivity to bolus arrival time (Massaro et al., 2013; Boudes et al., 2014), and requires further technical improvement. On the other hand, PC MRI is a promising technique that provides accurate measurement of global CBF and has been successfully used in health newborns recently to measure CBF (van Kooij et al., 2010; Benders et al., 2011; Varela et al., 2012; Jain et al., 2014; Liu et al., 2014).

Cerebral metabolic rate of oxygen (CMRO_2_), a measurement of cerebral energy consumption, is also an important physiological marker of brain function. It can be evaluated by several methods, including O-15 PET (Herscovitch et al., 1985; Altman et al., 1993; Ibaraki et al., 2008; Bremmer et al., 2011), near infrared spectroscopy (NIRS) (Skov et al., 1993; Elwell et al., 2005; Kusaka et al., 2014), calibrated functional MRI (fMRI) based techniques (Bulte et al., 2012; Gauthier and Hoge, 2012; Wise et al., 2013) and venous oxygenation-based techniques (Golay et al., 2001; Bolar et al., 2011; Qin et al., 2011; Guo and Wong, 2012; Xu et al., 2012; Liu et al., 2013). O-15 PET is rarely used in neonates due to radiation concern. NIRS requires the assumptions of the arteriovenous volume ratio and is difficult to determine the light penetration depth. Thus, although quick and bedside-accessible, NIRS measurements of CMRO_2_ are more limited to superficial brain tissues compared with deep brain tissues. For calibrated fMRI based methods, the long scanning time and the need to inhale special gas mixtures make it difficult for applications in newborns. In recent years, a few venous oxygenation-based MRI techniques have been proposed to measure CMRO_2_ in neonates (De Vis et al., 2014; Jain et al., 2014; Liu et al., 2014). T2-Relaxation-Under-Spin-Tagging (TRUST) MRI is one of the MRI techniques that measures venous oxygenation non-invasively (Lu and Ge, 2008; Xu et al., 2012) and has been validated in adults (Lu et al., 2012).

In this study, we will use the PC and TRUST MRI techniques to measure CBF, Yv, OEF and CMRO_2_ in neonatal patients with PWML, for the understanding of the perinatal pathophysiology of PWML. We will also assess the cerebral maturation of these neonates and evaluate its relationship with the hemodynamic parameters measured by MRI.

## Materials and methods

### Subjects

The study was approved by the Medical Ethics Committee of Shengjing Hospital of China Medical University and was granted a waiver of informed consent. A total of 51 neonates underwent MRI in Shengjing Hospital were included in this study between December 2015 and April 2016. Neonates who met the following criteria were excluded: severe hypoxia (1/5-min Apgar score <3); prenatal infection; hypoglycemia (blood glucose <60 mg/dl); encephalitis; cerebral intraparenchymal hemorrhage or subarachnoid hemorrhage; congenital anatomic or chromosomal anomaly; critical congenital heart disease. The MRI session of each neonate included the standard clinical sequences [anatomic T1- and T2 weighted MRI, diffusion weighted imaging (DWI)], followed by the PC and TRUST MRI scans. Based on the reading of the clinical anatomic images by radiologists (blinded to the PC and TRUST results), the neonates were categorized into two groups, the PWML group and the normal control group. Twenty-three newborns were categorized into the PWML group, including 14 males and 9 females. The gestational age (GA) at birth, postmenstrual age (PMA) at MRI scan and the birth weight were 34.00 (2.43) [median (interquartile range)] weeks, 35.14 (3.29) weeks and 2200 (860) g, respectively. There were 14 newborns with dyspnea after birth, eight preterm newborns had low birth weight, eight newborns had normal birth weight and one newborn had meconium aspiration and seizure. Among those newborns, eight newborns had cystic lesions. PWML contained four grades from I to IV. Cystic lesions belonged to grade IV of PWML. Dyspnea after birth, low birth weight and meconium aspiration are the common reasons leading to PWML.

Twenty-eight newborns were categorized into the control group, including 23 males and 5 females. The GA at birth, PMA at MRI scan and birth weight were 34.43 (6.43) weeks, 35.71 (5.36) weeks and 1,880 (1,645) g, respectively. There were nine preterm newborns with low birth weight. Neonates in the control group had normal results from umbilical cord blood gas analysis, liver function test, routine blood examination, and serum and ion analyses at birth. Their purpose of MR scans were for exclusions of abnormality of nervous system.

The clinical characteristics of the two groups are shown in Table 1.

**Table 1:** Clinical characteristics of the 51 infants.

**Characteristics**	**Infants in PWML group (*n* = 23)**	**Infants in control group (*n* = 28)**	**Z/χ^2^**	**Significance (*P*-value)**
No. of males (%)	14 (60.87)	23 (82.14)	−10.37	0.001^*^
Birth weight (g)	2,200 (860, 1,125–3,200)	1,880 (1,645, 1,140–5,250)	0.09	0.932
Gestational age at birth (weeks)	34.00 (2.43, 28.43–41.14)	34.43 (6.43, 26.89–41.29)	−0.43	0.670
PMA at MRI scan (weeks)	35.14 (3.29, 32.57–41.71)	35.71 (5.36, 29.57–45.43)	−0.86	0.389
Ya (%)	95.00 (0.00, 92.00–98.00)	95.00 (2.00, 90.00–99.00)	0.26	0.793
HCT (%)	45.60 (22.00, 29.80–65.10)	37.85 (11.95, 25.00–52.80)	1.97	0.049^*^
No. of low birth weight (<1,500 g) (%)	6 (26.09)	3 (10.71)	21.35	<0.001^*^

*Characteristics values are reported as frequency (percentage, the total range) or median (interquartile range, the total range) across subjects. P-values are based on chi-square test and Wilcoxon two-sample exact tests. Ya, arterial oxygen saturation; HCT, hematocrit; PMA, Postmenstrual age*.

* *P < 0.05*.

### General MRI protocol

All MRI scans were performed on a 3.0 T MR scanner (Intera Achieva, Philips Medical Systems, Best, the Netherlands) with an eight-channel, phased-array coil for reception. Newborns were well-fed, sedated immediately prior to imaging with nasogastric chloral hydrate (30–50 mg/kg), earplugs were inserted, and they were kept warm and monitored by a pediatrician throughout the scanning procedure.

The standard clinical protocols for radiological reading included T1-weighted spin echo imaging (repeat time (TR) = 200 ms, echo time (TE) = 2.3 ms, matrix = 224 × 162, field of view (FOV) = 180 × 150 × 89 mm^3^, section thickness = 5 mm), T2-weighted DRIVE CLEAR imaging (TR = 5,000 ms, TE = 80 ms, matrix = 240 × 135, FOV = 180 × 150 × 90 mm^3^, section thickness = 5 mm), echo-planar imaging (EPI) for DWI (TR = 3,500 ms, TE = 63 ms, section thickness = 5 m, matrix = 112 × 112, FOV = 180 × 180 × 89 mm^3^, b values 0 and 1,000 s/mm^2^). All images were acquired in the same axial plane.

### CBF measurement

**Figure 1:**
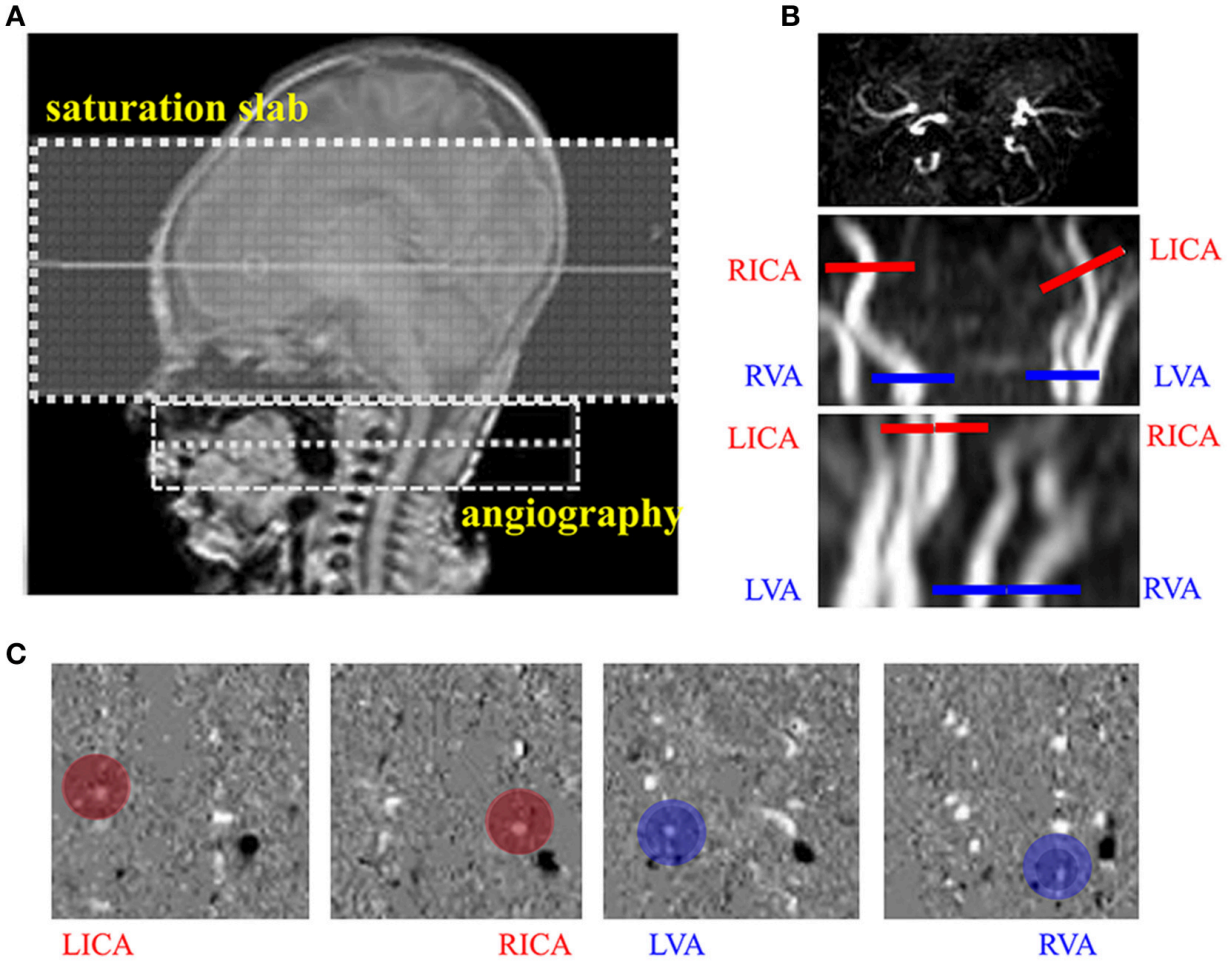
Measurement of cerebral blood flow (CBF) using phase contrast (PC) MRI. **(A)** Positioning of magnetic resonance angiography (MRA). **(B)** Positioning of PC MRI for the left internal carotid arteries (LICA), right internal carotid arteries (RICA), leftvertebral arteries (LVA) and right vertebral arteries (RVA) using the maximal-intensity-projection (MIP) images of the TOF angiogram. **(C)** Example velocity maps of the LICA, RICA, LVA, and RVA. Blood flow velocity was measured in the velocity map (red and blue circle).

For the CBF measurement by PC MRI, a time-of-flight (TOF) angiography was first performed to visualize the left internal carotid arteries (LICA), right internal carotid arteries (RICA), left vertebral artery (LVA) and right vertebral artery (RVA), which were the feeding arteries of the brain. The imaging slab was positioned to be centered at epistropheus (C2 vertebrate), with a 60-mm saturation slab above the imaging slab to suppress the venous signal (Figure 1A). Imaging parameters of the TOF angiography were: TR = 20 ms, TE = 3.45 ms, flip angle = 18°, FOV = 90 × 90 × 20 mm^3^, voxel size = 0.8 × 0.8 × 2 mm^3^, scan duration = 23.7 ms. Next, based on the maximal-intensity-projection (MIP) images of the TOF angiogram, four PC MRI scans were performed to measure blood flow, each targeting one of the four feeding arteries. The imaging slices were positioned to be centered on and perpendicular to the target arteries. The LICA and RICA slices were placed at the level of foramen magnum, and the LVA and RVA slices right below the level of C2 vertebrae to avoid the turning points (Figure 1B). PC MRI parameters were: single slice, voxel size = 0.5 × 0.5 × 3 mm^3^, FOV = 180 × 180 × 3 mm^3^, maximum velocity encoding = 20 cm/s, non-gated,2 averages, scan duration of each artery = 14.7 s. Using the above protocol, the total duration to obtain CBF in each newborn was approximately 1.5 min.

Three images were generated by PCMRI scans: an anatomic image, a magnitude image and a velocity map (Figure 1C). Data processing of PC MRI followed methods reported previously (Liu et al., 2014). A region of interest (ROI) was manually drawn by an experience researcher (P.L., >10-year experience) on the magnitude image by tracing the boundary of the targeted artery, and then applied to the velocity map to measure the flow velocity of the target artery. The velocity values from individual voxels within the ROI were integrated over the area of the ROI to yield the blood flux of each artery. To account for brain size differences, the unit volume CBF (in mL/100 g/min) was obtained by normalizing the total blood flux (in mL/min) of all four arteries to the brain parenchyma weight. The brain's parenchyma weight was converted from the total volume of gray matter and white matter (obtained from segmentation of the T2-weighted images) by assuming a parenchyma density of 1.06 g/ml (Herscovitch et al., 1985).

### Blood oxygen saturation measurement

**Figure 2:**
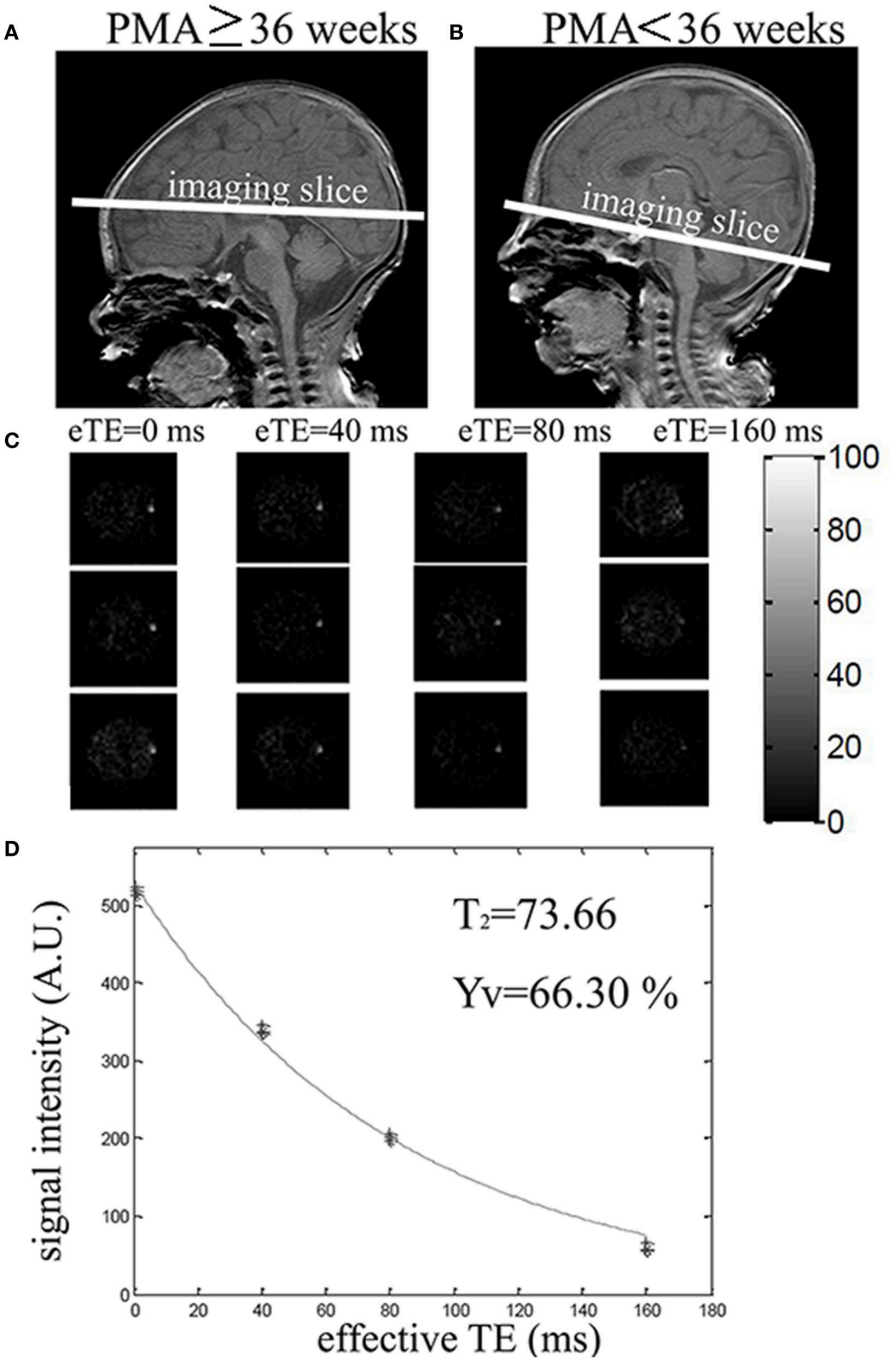
Positioning and measurement of Yv using T2-Relaxation-Under-Spin-Tagging (TRUST) MRI. **(A)** Positioning of TRUST MRI with PMA ≥ 36 weeks. **(B)** Positioning of TRUST MRI with PMA < 36 weeks. **(C)** Difference images between control and label for different effective TEs (eTEs): 0, 40, 80, and 160 ms. **(D)** Monoexponential fitting of the signal intensity in target venous sinus as a function of eTE yielded the venous blood. T2 was then converted into Yv via a calibration plot.

Ya was assessed using a pulse oximetry, which had an optical sensor attached to the toe of the newborn, while Yv was measured by TRUST MRI (Liu et al., 2014). The TRUST sequence utilizes the spin labeling scheme to isolate pure venous blood signal, and modulates T2-weighting of the venous signal to obtain venous T2, which can then be converted to venous oxygenation via the T2-Yv calibration (Liu et al., 2014, 2016). Due to the age-related variation in blood flow velocity and vessel size in newborns, two imaging locations were used to obtain >20% labeling efficiency of the TRUST scan. If PMA ≥36 weeks, imaging slices were positioned parallel to the intercommissural line with a 10 mm distance from the top of the sinus confluence to measure Yv in the superior sagittal sinus (Figure 2A). If PMA < 36 weeks, imaging slices were positioned parallel to the intercommissural line and below the sinus confluence and to measure Yv in the transverse or sigmoid sinuses (Figure 2B). The difference between control and label images yielded blood signal from the target vein, which was modulated with different T2-weighting using four different TEs: 0, 40, 80, and 160 ms, which were called effective TE (eTE) (Figure 2C). Monoexponential fitting of the signal intensity in target venous sinus as a function of eTE yielded the Carr–Purcell–Meiboom–Gill (CPMG) T_2_ of the venous blood (Figure 2D). T2 was then further converted into Yv via a calibration plot (Liu et al., 2016). The thickness of the labeling slab was 80 mm. The following parameters were used for TRUST scan: TR = 3,000 ms, inversion time (TI) = 1,022 ms, FOV = 160 × 160 × 5 mm^3^, matrix size = 64 × 61, SENSE factor = 3, voxel size = 2.5 × 2.5 × 5 mm^3^ and τCPMG = 10 ms. Three pairs of control and labeled images were scanned for each eTE and the total scan duration was 72 s.

Following the analysis described previously (Liu et al., 2014), the TRUST data were motion corrected using the software Statistical Parametric Mapping (SPM2, University College, London, UK). Pairwise subtraction between control and labeled images yielded the difference images for each eTE. A coarse ROI was manually drawn to include the targeted vein in the area. Six voxels with the largest signal intensity within the ROI were chosen to calculate venous blood signal. The averaged venous signal intensity for each eTE was then fitted to a monoexponential curve to obtain a T2 value (Figure 2D). T2 was converted to Yv via a calibration plot using the hematocrit (HCT) values from routine blood examination of the neonates.

### Calculation of CMRO_2_ and OEF

CMRO_2_ and OEF values were calculated using the following equations (Liu et al., 2013, 2016):

(1) $$\begin{array}{r} \text{CMR}{\text{O}}_{2}=\text{CBF}\times \left(\text{Ya}-\text{Yv}\right)\times \text{Ca}, \end{array}$$

(2) $$\begin{array}{r} \text{OEF}=\left(\text{Ya}-\text{Yv}\right)/\text{Ya}\times 100\text{\%}, \end{array}$$

where the unit of CMRO_2_ is μmol O_2_/100 g/min. Ca is the capacity of blood to carry O_2_ in a HCT unit. Based on the data from physiology literature, Ca = 897 μmol O_2_/100 ml when HCT is 0.44 (Guyton and Hall, 2005).

### Evaluation of cerebral maturation

Neonatal cerebral maturation was assessed using the total maturation score (TMS) validated by different groups (Childs et al., 2001; Ramenghi et al., 2007). TMS utilizes four parameters in the scoring system on T1, 2-weighted images: two phenomena undergoing progressive maturation (myelination [M], and cortical folding [C]), and two structures undergoing progressive involution (glial cell migration bands [G], and bands of migrating glial cells [B]). M ranges from 1 to 7 (M1–M7), C ranges from 1 to 6 (C1–C6), G ranges from 1 to 4 (G1–G4) and B ranges from 1 to 4 (B1–B4). The sums of the four parameters yield the TMS. C and G were observed at the plane of the interventricular foramen. Detailed scoring criteria are shown in Table 2. To minimize the inter-rater dependence of TMS, MR images were reviewed independently by two experienced pediatric radiologists (YQ and XW, both with >10-year experience). The mean values of the assessments by YQ and XW were calculated and used in the statistical analysis.

**Table 2:** Scoring system of total maturation score to assess four parameters of cerebral maturation in newborns.

**Total maturation score (TMS)**	**Parameter**	**Score**	**Characteristic of T1 or 2-weighted images**
Progressive maturation	Myelination (M) on T2-weighted images	M1	Myelination evident in brainstem, cerebellar peduncle, inferior colliculus, cerebellar vermis
		M2	M1+ Subthalamic nuclei, globus pallidus, ventrolateral thalamus
		M3	M2+ Caudal portion of the posterior limb of the internal capsule (PLIC)
		M4	M3+ Complete PLIC
		M5	M4+ Optic radiation
		M6	M5+ Corona radiate
		M7	M6+ Anterior limb of internal capsule
	Cortical infolding (C) on T1-weighted images	C1	Frontal and occipital cortex completely smooth, insula wide open; thin bright cortical rim, generally low-intensity white matter (WM)
		C2	Frontal cortex still very smooth, some sulci evident in occipital cortex; insula still wide with almost smooth internal surface; WM low intensity
		C3	Frontal and occipital cortex similar number of convolutions; frontal sulci still quite shallow; internal surface of insula more convoluted; WM still low intensity
		C4	Frontal and occipital cortex folded and rich in sulci; frontal sulci obvious along interhemispheric fissure; occipital WM separated into strands by deeper sulci; Insula more convoluted and infolded; WM still slightly low intensity
		C5	Frontal and occipital WM separated into strands by deeper sulci; insula completely infolded; WM still distinguishable from gray matter
		C6	As C5 but WM now isointense with gray matter
Progressive involution	Germinal matrix (G) on T2-weighted images	G1	Matrix seen in posterior horn, at caudothalamic notch (CTN) and anterior horns of lateral ventricles
		G2	Matrix evident at CTN and anterior horns only
		G3	Matrix at anterior horns alone
		G4	No matrix evident
	Bands of migrating glial cells (B) on T2-weighted images	B1	Broad band with additional narrower bands
		B2	Broad band alone
		B3	Narrow band alone
		B4	No bands seen

### Statistical analysis

Continuous outcomes were summarized using medians and interquartile ranges and categorical outcomes using frequencies and percentages. Given the relatively small number of PWML group (*N* = 23) and controls (*N* = 28) in this study, summary measures were compared between groups using Wilcoxon two-sample exact tests (for continuous variables) and Chi-Square tests (for categorical variables). The inter-rater variation in the TMS determined by YQ and XW was estimated using the Intraclass Correlation Coefficient (ICC). For values ranging from 1.0 to 0.81, the reliability was considered excellent; from 0.80 to 0.61, very good; from 0.60 to 0.41, good; from 0.40 to 0.21, reasonable and, finally, from 0.20 to 0.00, poor.

To evaluate the group difference with the consideration of age effect, multi-linear regression analyses were performed with the measured physiological parameter (Yv, OEF, CMRO_2_, CBF, and brain volume, respectively) as dependent variable, and group (PWML or control) and PMA at MRI scan as independent variables. Similar analyses were also performed with group category and TMS as independent variables. In addition, stepwise regression analysis was performed to evaluate the effect of HCT on CMRO_2_, where CMRO_2_ was the dependent variable, PMA (and TMS), HCT and group were independent variables.

ROC used to determine whether Yv, OEF, CMRO_2_, CBF and brain volume faciliated the diagnosis of PWML. Classing the accuracy of a diagnostic test in the traditional academic point system: excellent = 0.9–1.0, good = 0.8–0.9, fair = 0.7–0.8, poor = 0.6–0.7, fail = 0.5–0.6 (significance was assessed at *P* < 0.05, SPSS 22).

## Results

### Comparison between the two groups

The demographic information of the two groups is shown in Table 1. There were no significant differences in birth age, scan age and Ya between the two groups, but sex, HCT values and number of low birth weight showed group difference. Representative images of the PC and TRUST MRI scans were shown in Figures 1, 2, respectively.

**Figure 3:**
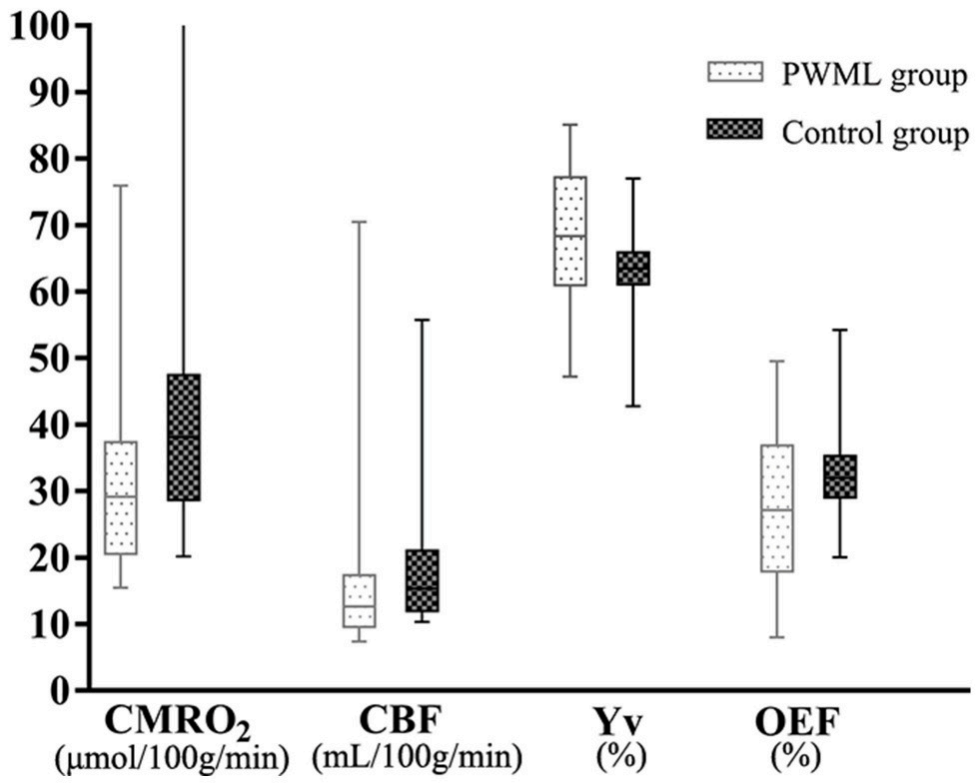
CMRO2-, CBF-, Yv-, and OEF-data for 28 control and 23 PWML infants. The median, interquartile range, minimum and maximum values for the CMRO_2_, CBF, Yv, and OEF of the two groups were shown; The CMRO_2_, CBF, Yv, and OEF in PWML group differed significantly from the parameters in control group (*Z* = −2.33, *P* = 0.020; *Z* = −2.22, *P* = 0.027; *Z* = 2.53, *P* = 0.012; *Z* = −2.38, *P* = 0.018). One newborn in the control group with an outlier of CMRO_2_ value was 144.25 μmol/100 g/min.

Table 3 showed the median, interquartile ranges and ranges of CMRO_2_, CBF, Yv, OEF, brain volume and TMS of the two groups, as well as their outcomes of comparison. Significant differences between PWML and control groups were found in CMRO_2_ (*P* = 0.020), CBF (*P* = 0.027), Yv (*P* = 0.012), OEF (*P* = 0.018), TMS (*P* = 0.011), M scores (*P* < 0.001), C scores (*P* = 0.016), and B scores (*P* = 0.018). Yv and B scores were higher and other measurement values were lower in PWML group. The median, interquartile range, minimum and maximum values for CMRO_2_, CBF, Yv, and OEF from both groups, were shown by means of boxplots in Figure 3.

**Table 3:** Yv, OEF, CBF, CMRO_2_, brain volume and TMS in PWML group and control group.

**Parameter**	**Infants in PWML group (*n* = 23)**	**Infants in control group (*n* = 28)**	**Z**	**Significance (*P*-value)**
Yv (%)	68.70 (16.30, 48.00–86.00)	63.85 (4.80, 43.00–77.00)	2.53	0.012^*^
OEF (%)	27.53 (18.94, 8.00–50.00)	32.35 (6.26, 20.00–55.00)	−2.38	0.018^*^
CBF (mL/100 g/min)	12.63 (7.83, 7.35–70.46)	15.35 (9.13, 10.31–55.75)	−2.22	0.027^*^
CMRO_2_ (μmol/100 g/min)	29.11 (16.80, 15.49–75.94)	38.09 (18.84, 20.15–144.25)	−2.333	0.020^*^
Brain volumes (mL)	280.35 (48.80, 229.90–366.49)	276.84 (84.82, 134.91–435.91)	0.25	0.806
TMS	9.00 (2.50, 6.00–16.00)	11.00 (5.00, 5.50–16.00)	−2.53	0.011^*^
M scores	2.00 (0.00, 2.00–6.00)	3.00 (4.00, 1.50–6.00)	−3.62	< 0.001^*^
C scores	3.00 (1.00, 2.00–5.00)	4.00 (1.75, 2.00–5.50)	−2.40	0.016^*^
G scores	2.00 (1.00, 1.00–4.00)	2.00 (0.88, 1.00–3.50)	−1.34	0.179
B scores	2.00 (1.00, 1.00–4.00)	1.00 (0.75, 1.00–3.00)	2.37	0.018^*^

*Parameter values are reported as median (interquartile range, the total range) across subjects. P-values are based on Wilcoxon two-sample exact tests. CBF, cerebral blood flow; Ya, arterial oxygen saturation; Yv, venous oxygen saturation; OEF, oxygen extraction fraction; CMRO_2_, cerebral metabolic rate of oxygen; TMS, total maturation score; M, Myelination; C, Cortical folding; G, Glial cell migration bands; B, Bands of migrating glial cells*.

* *P < 0.05*.

The inter-rater ICC value of TMS was 0.987. The reliability was considered excellent.

### Effects of group and PMA on measured physiological parameters

**Figure 4:**
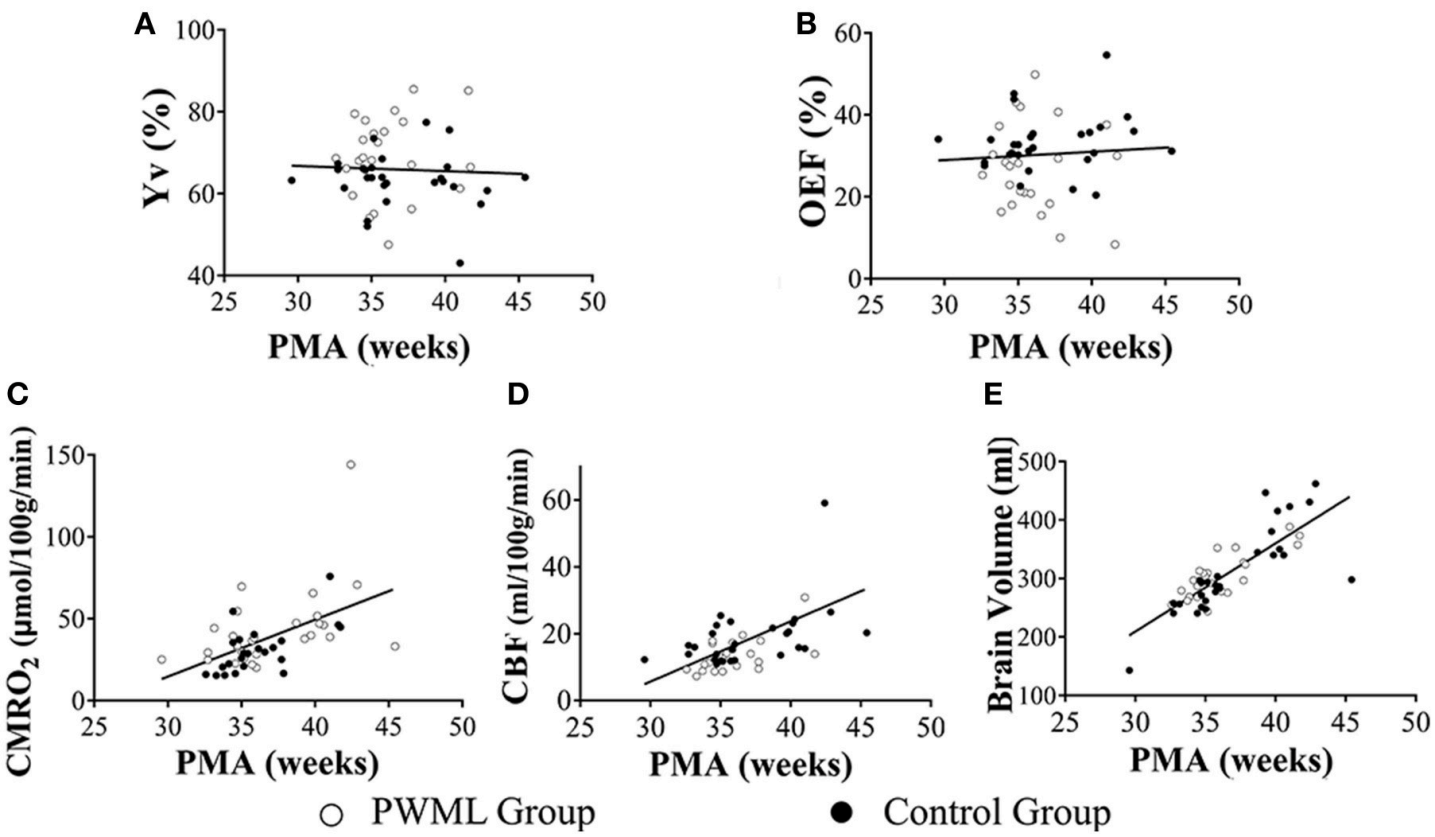
Scatter plots of the physiological parameters as a function of PMA. Yv **(A)** and OEF **(B)** had no dependence on PMA (*P* = 0.993, *P* = 0.862, respectively). CMRO_2_ **(C)**, CBF **(D)** and brain volume **(E)** demonstrated a significant increase with PMA (*P* < 0.001). Filled symbols indicate control neonates, and unfilled symbols indicate PWML patients.

Figure 4 showed the scatter plots of the measured physiological parameters as a function of PMA at MRI scan. Considering all neonates together, the multi-linear regression analysis showed that both Yv and OEF had significant correlation with group (*P* = 0.022 and *P* = 0.028, respectively), while had no dependence on PMA at MRI scan (*P* = 0.993 and *P* = 0.862, respectively). CMRO_2_ demonstrated a significant increase with PMA at MRI scan (*P* < 0.001), but had no significant relationship with group (*P* = 0.093). Similarly, a significant increase in CBF and brain volume were found with PMA (*P* < 0.001), but not with category (*P* = 0.748). Stepwise regression analysis confirmed that CMRO_2_ was significantly correlated with PMA (*P* < 0.001), but not with HCT (*P* = 0.999) and group (*P* = 0.979).

### Effects of group and TMS on measured physiological parameters

**Figure 5:**
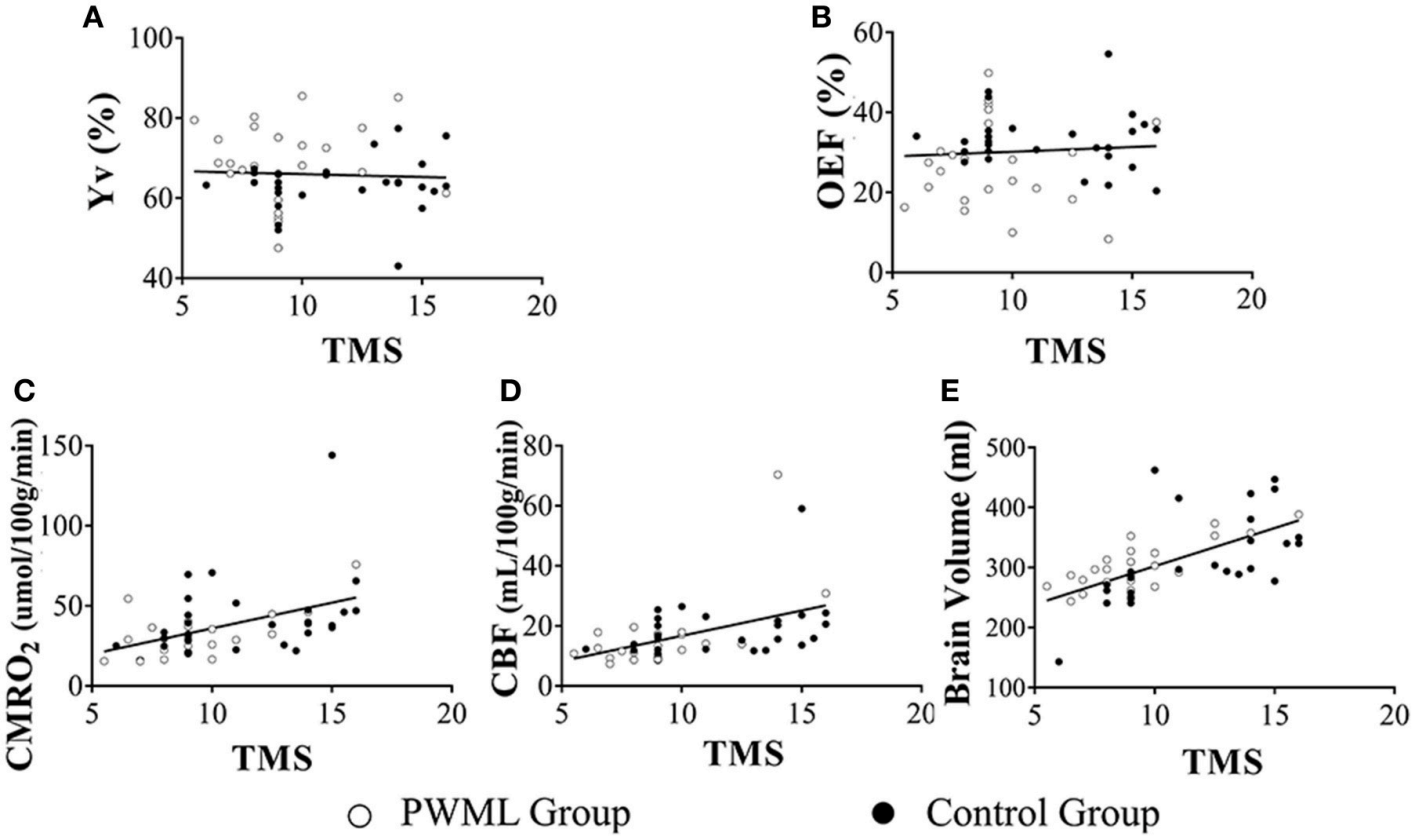
Scatter plots of the physiological parameters as a function of TMS. Yv **(A)** and OEF **(B)** had no dependence on TMS (*P* = 0.582, *P* = 0.747, respectively).CMRO_2_ **(C)** (*P* = 0.005), CBF **(D)** (*P* = 0.001) and brain volume **(E)** (*P* < 0.001) increased significantly with TMS. Filled symbols indicate control neonates, and unfilled symbols indicate PWML patients.

Figure 5 showed the scatter plots of the measured physiological parameters as a function of TMS. The multi-linear regression analysis showed that both Yv and OEF had significant correlation with group (*P* = 0.019 and *P* = 0.028, respectively), while had no dependence on TMS (*P* = 0.582 and *P* = 0.747, respectively). CMRO_2_ (*P* = 0.005), CBF (*P* = 0.001) and brain volume (*P* < 0.001) increased significantly with TMS, but all had non-significant relationship with group (*P* = 0.353, *P* = 0.521, *P* = 0.089). Stepwise regression analysis also showed that CMRO_2_ was significantly correlated with TMS (*P* = 0.001), but not with HCT (*P* = 0.999) and group (*P* = 0.866).

### ROCs of the physiological parameters for the diagnosis of PWML

**Figure 6:**
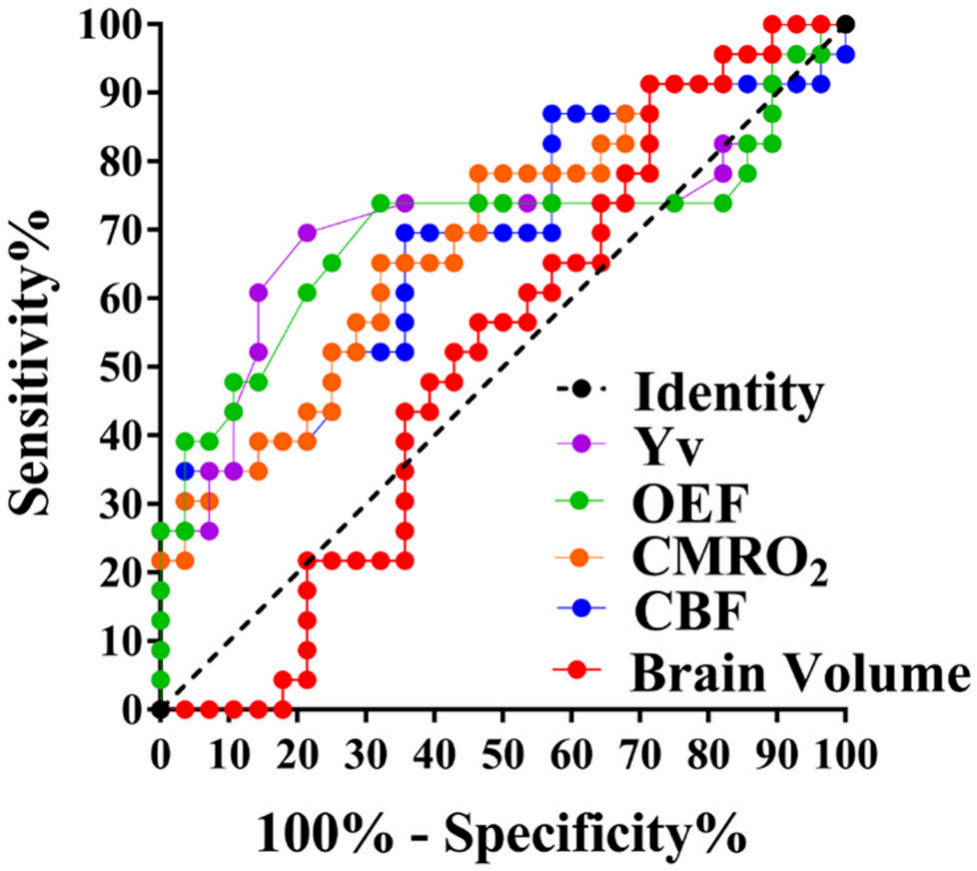
ROCs of Yv, OEF, CMRO_2_, CBF, and brain volume. AUC (Yv) = 0.71, AUC (OEF, CMRO_2_, CBF, and brain volume,) = 0.70, 0.69, 0.68, and 0.52, respectively.

The ROCs of Yv, OEF, CMRO_2_, CBF and brain volume were shown in Figure 6. Their areas-under-curve (AUC) were 0.71 (confidence interval (C.I.): 0.55–0.86), 0.70 (0.54–0.86), 0.69 (0.54–0.84), 0.68 (0.53–0.83), and 0.52 (0.36–0.68), respectively, suggesting a fair diagnostic value of Yv and OEF.

## Discussion

In this study we used the PC and TRUST MRI techniques to measure CBF, Yv, OEF, and CMRO_2_ in neonates with PWML and compared their values with those measured in healthy neonates. Our results demonstrated that newborns with PWML had lower values of CMRO_2_, CBF, OEF, and TMS, but higher value of Yv than the healthy controls. Taken into account the effect of age and brain maturation, Yv and OEF still showed a significant difference between the two groups. CMRO_2_, CBF and brain volume were correlated with age and cerebral maturation score.

### Comparison of the physiological parameters with previous literature

Using various techniques including PET (Altman et al., 1993), MRI (Liu et al., 2013, 2014), and NIRS (Skov et al., 1993; Elwell et al., 2005), the measurements of CMRO_2_, CBF, OEF, and Yv in infants with asphyxia, ventilation dependence, and respiratory distress syndrome were reported (detailed results in Table 4). Results from all previous studies found that unhealthy infants had lower CMRO_2_, CBF and OEF, and higher Yv values compared with healthy infants. For healthy neonates, our OEF and CMRO_2_ values were consistent with the previous MRI report by Liu et al. (2014), but slightly higher than the values reported from De Vis et al. (OEF = 49%, CMRO_2_ = 30 μmol/100 g/min) (De Vis et al., 2014). Our results from the PWML group showed some difference with the PET study by Altman et al. (1993), (OEF = 16.6 %, CBF = 21.6 mL/100 g/min, CMRO_2_ = 21.4 μmol/100 g/min), but were within the range of values in diseased neonates measured with MRI (De Vis et al., 2014; Jain et al., 2014) and NIRS (Skov et al., 1993; Elwell et al., 2005) methods. The study from Altman group was primarily focused on newborns with severe brain injuries or those that needed extracorporeal membrane oxygenation (ECMO). The degree of severity of the brain injuries might be the reason of this difference. It is also possible that the sedation drug used in this study, chloral hydrate, may contribute to the difference with other studies. Although there is no direct evidence in human, animal studies have suggested that chloral hydrate might lead to higher CBF and lower glucose metabolism (Grome and McCulloch, 1981; Uematsu et al., 2009). Other factors that may lead to the variations across studies include subject age, sample size, whether there was oxygen support during study, as well as the techniques used for measurement. The TRUST MRI technique used in this study has been validated in adults previously (Lu et al., 2012). Yv measured with this technique is relatively robust to variations in brain size and blood flow velocity due to use of spin labeling scheme and flow-insensitive T2 preparation. In addition, a neonatal-specific T2-oxygenation calibration plot was applied to improve the accuracy of Yv measurement using TRUST MRI (Liu et al., 2016). Overall, the results in the present study are generally in good agreement with previous literature. We believe that PC MRI and TRUST MRI are reliable and efficient techniques for observing the changes of cerebral oxygen supply, and oxygen consumption and metabolic rates in newborns with PWML.

**Table 4:** Comparison of the arterial oxygenation (Ya), venous oxygenation (Yv), oxygen extraction fraction (OEF), cerebral blood flow (CBF), and cerebral metabolic rate of oxygen (CMRO_2_) obtained from this study with that of previous reports.

**Study**	**Method**	**Number of subjects and condition**	**PMA (weeks)**	**Ya (%)**	**Yv (%)**	**OEF (%)**	**CBF (mL/100 g/min)**	**CMRO_2_ (μmol/100 g/min)**
Altman	PET	10 HIE, RDS, ECMO	35.1 ± 6.2	–	21.6 ± 21.0	16.6 ± 13.2	21.6 ± 21.1	21.4 ± 16.4
De vis	MRI	10 PMA at term 9 HIE	39 38	97 ± 1 96 ± 3	52 ± 12 65 ± 13	49 ± 12 32 ± 12	14 ± 3 12 ± 4	30 ± 6 24 ± 12
Liu	MRI	10 healthy	37.4 ± 2.6	95.8 ± 2.2	62.6 ± 8.3	33.3 ± 2.7	13.4 ± 4.2	38.3 ± 17.7
This study	MRI	28 healthy 23 PWML	35.71 (5.36) 35.14 (3.29)	95 (2) 95 (2)	63.85 (4.80) 68.70 (16.30)	32.35 (6.26) 27.53 (18.94)	15.35 (9.13) 12.63 (7.83)	38.09 (18.84) 29.11 (16.80)
Elwell	NIRS	9 ventilatory support	29.2 ± 5.3	–	–	–	–	45.9 ± 12.3
Skov	NIRS	10 asphyxiated (term) 22 RDS (preterm)	38.8 ± 1.4 29.8 ± 2.6	94 ± 7 96.5 ± 5	67.3 ± 9.4 53.4 ± 15.4	28.4 ± 0.3 44.6 ± 2.1	26.5 ± 17.9 11.9 ± 5.2	62.6 ± 35.8 44.7 ± 17.9

*Parameter values are reported as median (interquartile range) in this study and mean ± SD in previous reports. PMA, postmenstrual age at MRI scan; HIE, Hypoxic ischemic encephalopathy; RDS, Respiratory distress syndrome; ECMO, Extracorporealmembrane oxygenation*.

### Physiologic changes in PWML

Our results demonstrated that both OEF and Yv had significant dependence on groups. Specifically, Yv was higher and OEF was lower in the PWML group. These differences are likely to be due to the effect of hypoxia. Hypoxia is one of the primary causes of both PWML and HIE. In this study, 14 newborns in the PWML group had reported perinatal hypoxia or breathing difficulties (60.87%). Hypoxia is known to induce neuronal degeneration and necrosis and severehypoxia can lead to cystic lesions (Counsell et al., 2003; Volpe, 2003; Back et al., 2006; Robinson et al., 2010). Consequently, oxygen consumption in the brain would be reduced, leading to increased Yv and decreased OEF. Results of De Vis et al. (2014) and Shi et al. (2012) demonstrated similar effect of hypoxia in newborns with HIE.

Most PWML cases can be clearly recognized on MRI during the first 2 weeks. However, due to absorption, PWML become difficult to identify as lesions become smaller, less, and their T1-hyperintensity disappears. Moreover, brain volume as a quantitative anatomic marker failed to differentiate between the PWML and normal controls in our study, even though preterm newborns with low birth weight or extremely low birth weight have higher incidence of PWML. On the other hand, we observed a fair diagnostic value of Yv and OEF in PWML, despite the variety of severity in our PWML group. Therefore, Yv and OEF could be valuable physiological biomarkers that may assist with the diagnosis of pathophysiological abnormality.

We found that CMRO_2_ in the PWML group were lower than the control group (27%; Figure 3), although didn't reach a significant level after accounted for the group differences (*P* = 0.093). The lack of significance could be due to the relatively small sample size, as well as confounding factors such as respiratory and circulatory.

### Relationships with age and cerebral maturation

Results from the present study, as well as from studies by De Vis et al. (2014) and Liu et al. (2014), all found that both CMRO_2_ and CBF had significant correlation with age. Since PMA is an indirective parameter of brain development, CMRO_2_ and CBF are therefore associated with brain development. In this study, we further assessed brain maturation by TMS considering four indices: myelination, cortical folding, glial cell migration and germinal matrix tissue. Myelination begins approximately 20 weeks in the fetus, 24–25 weeks in the dorsal thalamus and globus pallidus, and 35–36 weeks in striatum. The positive correlation between CBF and TMS, and between CMRO_2_ and TMS suggested that as myelination progresses, the blood supply and oxygen metabolism in the brain increase to meet the escalating energy demand.

Previously De Vis et al. (2014) reported that OEF and Yv were positively correlated with PMA. However, we did not observe any age-dependence between OEF and Yv. These differences may be due to the relatively small sample size in the present study, and therefore larger sample sizes will be needed in the future.

### HCT differences between the two groups

The HCT values were higher in the PWML group compared to the control group (*P* = 0.049), similar to a previous study in newborns with HIE (De Vis et al., 2014). HCT indicates the number of red blood cells which predicts the distribution of oxygen (Gould and Linninger, 2015), and is the primary factor used to determine blood viscosity. Asphyxia induces hypoxia in cells, and decreases adenosine triphosphate (ATP) production as well as pH values and blood flow velocity, all of which lead to decreased erythrocyte deformability and increased blood viscosity. Thus, the increased HCT values in PWML and HIE newborns were consistent with the pathophysiology of asphyxia. Additionally, gender may also affect the HCT values. In our control group there were 5 females and 23 males, likely to be due to gender discrimination happened occasionally in China. Although group difference was found in HCT, our results suggested that HCT has no effect in CMRO_2_ measurements.

## Conclusions

In the present study, we demonstrated the feasibility of quantifying CMRO_2_, CBF, Yv and OEF in neonates with PWML using non-invasive MRI methods. Lower cerebral oxygen consumption was found in the PWML neonates. Our results suggested that physiological parameters such as Yv and OEF may be helpful for the diagnosis of PWML. The positive correlation between CBF and TMS, and between CMRO_2_ and TMS suggested that as myelination progresses, the blood supply and oxygen metabolism in the brain increase to meet the escalating energy demand.

## Author contributions

XW, HL, PL, and YQ participated in conceiving and designing of the idea. PL and ZL contributed to providing the post-processing assistant. HL, PL, and YQ contributed to analyzing the experiment results. YQ also contributed to drafting and editing of the manuscript. HL, PL, XW, and YQ also participated in critically revising the paper. All authors have read and approved the final manuscript for publication.

### Conflict of interest statement

The authors declare that the research was conducted in the absence of any commercial or financial relationships that could be construed as a potential conflict of interest.
